# Relatives' experiences of brief admission in borderline personality disorder and self‐harming behaviour

**DOI:** 10.1002/nop2.1487

**Published:** 2022-11-20

**Authors:** Sally Hultsjö, Hanna Rosenlund, Lisa Wadsten, Rikard Wärdig

**Affiliations:** ^1^ Department of Psychiatry Ryhov County Hospital Jönköping Sweden; ^2^ Division of Nursing and Reproductive Health, Department of Health, Medicine and Caring Sciences Linköping University Linköping Sweden

**Keywords:** brief‐admission, experiences, interviews, qualitative research, relatives

## Abstract

**Aim:**

The aim of this study is to describe experiences of brief admission (BA) of people with borderline personality disorder and self‐harming behaviour, from the perspective of their relatives.

**Design:**

A descriptive qualitative design was chosen.

**Methods:**

Twelve relatives of people with borderline personality disorder and self‐harming behaviour who had access to BA were interviewed. Data were analysed with qualitative conventional content analysis.

**Results:**

One overarching category: Hope for the future and three categories occurred: Breathing space, Personal responsibility and Structure. BA created hope for the future and the relatives appreciated that BA is a freer and easily accessible form of care that enables help at an early stage, compared with usual care. When BA functions, the structure and pre‐determined days of care give relatives a breathing space, and the uncertainty diminishes for the children, as the parent can still be present during inpatient care. The lack of places was described as a disadvantage of BA.

**What does this paper contribute to the wider global clinical community?**

- Due to the psychological suffering, the great amount of responsibility and high burden experienced by relatives there is a need to explore new recommendations about clinical management from the viewpoint of relatives of individuals with BPD and self‐harming behaviour.
- As the structure and pre‐determined days of care given during brief admission give the whole family a breathing space, and better pre‐requisites for a functional everyday life, the concept could have benefits also for other psychiatric diagnoses.

## 1 INTRODUCTION

Brief admission (BA) is a relative new nursing crisis intervention offered to patients with borderline personality disorder (BPD) and self‐harming behaviour in Sweden, Denmark, Norway and the Netherlands (Helleman et al., 2014a, 2014b; Helleman, Goossens, et al., 2018; Thomsen et al., 2018). BA is complementary to regular admissions and can be used in an acute crisis to promote the constructive self‐regulation of emotions and reduce stress and anxiety, thereby preventing self‐harm (Helleman et al., 2014a) and reducing long‐term admissions (Eckerström et al., 2020). In contrast to regular psychiatric inpatient care where the physician has a gatekeeper function, BA is a patient‐initiated and self‐planned admission in psychiatric inpatient care limited in time. The patient plans the care content in advance. No previous studies concerning BA have been found from the perspective of relatives. Relatives of people with BPD are of great importance in the recovery process and their experiences are an important source of knowledge (Juntunen et al., 2018). Family involvement is a key to reach long‐lasting positive effects on mental health and family should be recognized as important members of the healthcare team. The term “person‐centred care” alone may not adequately capture the importance of the family (Feinberg, 2014). Thus, mental health services should be based on a person and family‐centred care, in which it is important to listen to both the patient and the relatives' voices to gain a more comprehensive understanding (Millenson et al., 2016).

## 2 BACKGROUND

The lifetime prevalence of BPD in the general population worldwide ranges from 1 to nearly 6 percent (Gunderson et al., 2018; Tyrer et al., 2010). In the mental healthcare system, BPD is one of the most common psychiatric diagnoses, and between 15%–28% of the patients are affected (Gunderson et al., 2018). BPD is a severe mental disorder characterized by chronic feelings of emptiness, difficulties controlling anger, uncertainty about one's own identity and a pattern of unstable and intense interpersonal relationships (American Psychiatric Association, 2013; Brickman et al., 2014; Gunderson et al., 2018). Nearly 65%–80% of those with BPD are also engaged in some form of self‐harm, with the consequence of repeated attempts at suicide (Brickman et al., 2014; Tyrer et al., 2010). Among Swedish adolescents aged 15–17 years, 56% reported at least one episode of self‐harm during the last year and nearly 7% met suggested DSM‐5 criteria for a potential non‐suicidal self‐injury (Zetterqvist et al., 2013). Among adults, 4%–5% have a history of self‐harm (Koyanagi et al., 2015). Although self‐harming behaviours tend to decrease over time, they usually leave persistent and severe social disabilities, with negative effects on life events (Gunderson et al., 2018). BPD is associated with a range of poor outcomes, including interruptions in education, and increased unemployment and family conflicts (Wertz et al., 2020). Alltogether, this leaves a high burden on both patients and their relatives (Bohus et al., 2021). Compared with relatives of people with other mental disorders, relatives of people with BPD show a higher degree of grief (Bailey & Grenyer, 2014), and many fear their close one will, due to self‐harming behaviour balancing in between life and death, find it hard to take care of themselves (Ekdahl et al., 2011). Relatives often take a great amount of responsibility and are important social support in the society for people with BPD (Sharma et al., 2016). Due to the psychological suffering (Gunderson et al., 2018) and the high burden experienced by relatives (Bailey & Grenyer, 2014), there is a need for effective treatments (Gunderson et al., 2018). Options vary greatly between different countries, but first‐line treatment for people with BPD includes some form of psychological intervention (Storebø et al., 2020). Dialectical behavioural therapy appears to be a valuable treatment in reducing both self‐harm and suicidal ideation (Kothgassner et al., 2021). Only a few of all patients with BPD are cared for in inpatient care, and then usually for suicidal behaviour (Hawton et al., 2012). In this context, there is a lack of knowledge‐based methods and there is a contradiction in admissions recommendations. BA is a nursing crisis intervention aimed at reducing long hospitalizations, preventing self‐harm and encouraging the patient's autonomy, self‐esteem and influence over care and treatment (Eckerström et al., 2020). A contract is designed according to the individual patient's needs in collaboration between the patient, outpatient staff and nurse in the inpatient ward. The contract contains a care and crisis plan and is shaped according to the patient's wishes. During BA, no contact with a physician is allowed (Eckerström et al., 2019). The opportunity to recover with the help of the department's routines and structure is mentioned as one of the most important parts of BA (Eckerström et al., 2020). The pre‐determined time for hospitalization of a maximum of 3 days is considered helpful as it minimizes dissatisfaction in connection with discharge (Ellegaard et al., 2017). BA offers the patient an opportunity to receive help at an early stage, so that emergency care and long hospitalizations can be avoided. BA further helps to shift the focus from the patient's difficulties to the patient's resources (Eckerström et al., 2019). Nurses experience that BA promotes the nurse–patient relationship and inspires security and continuity (Eckerström et al., 2019). Consumption of care in inpatient care for patients with severe self‐harming behaviour does not seem to decrease among BA consumers, but reduces self‐harm and number of days in compulsory care (Strand & von Hausswolff‐Juhlin, 2015; Westling et al., 2019). Patients described BA as being primarily comforting and empowering, especially given the fact that they can use it when needed (Eckerström et al., 2020); and it provides conditions to maintain valuable daily activities and relationships (Enoksson et al., 2021). Previous research has generated results that indicate that BA is effective and helpful from the perspectives of patients (Eckerström et al., 2020; Enoksson et al., 2021; Helleman, Goossens, et al., 2018) and healthcare staff (Arnold et al., 2021; Eckerström et al., 2019). To our knowledge, no previous study explores relatives' experiences of BA. Consumers with BPD and their relatives feel excluded and experience stigma and low quality care in traditional mental health services (Barr et al., 2020). They further experience that mental health care focuses on rapid treatments, which are not helpful and, after discharge, that responsibility depends entirely on them (Ekdahl et al., 2011), which in turn leads to a mistrust in psychiatric care. Thus, considering the perspectives of relatives is of importance when implementing new interventions in mental health services (Grenyer et al., 2017). Relatives want to be involved and recognized as a resource in the care of their close one (Weimand et al., 2013). Exploring relatives' experiences of BA can contribute to a deeper knowledge useful for mental health care in order to reach a person and family‐centred care (Feinberg, 2014; Millenson et al., 2016). Thus, the aim of the study was to describe experiences of (BA) for people with borderline personality disorder and self‐harming behaviour, from the perspective of their relatives.

## 3 METHODS

### 3.1 Design

A qualitative, explorative study design was chosen, as it provides new insights and increases the researcher's understanding of a particular phenomenon (Patton, 2015). Semistructured interviews were applied, as they encourage participants to express their experiences, though in a given frame. Conventional content analysis was used for analysis (Hsieh & Shannon, 2005). Conventional content analysis is useful when the purpose of the study is to study a phenomenon in an area where other research is sparse (Hsieh & Shannon, 2005). The research process and the presentation have followed the COREQ guidelines (Tong et al., 2007).

### 3.2 Sample and setting

Participants were recruited from a region containing three separate outpatient psychiatric clinics that worked in collaboration with their respective inpatient psychiatric clinics to implement BA for patients with BPD and self‐harming behaviour. First, the head of the department gave the research team written permission to conduct the study. After permission was received, healthcare staff working with BA were contacted to help identify patients diagnosed with BPD and self‐harming behaviour who had been assigned to BA, regardless of when and how often they have used BA, all patients have constant access even though the majority did not use BA at the moment. The inclusion criterion for being invited to participate in the study was being a relative of a patient diagnosed with BPD and self‐harming behaviour, with a signed BA contract. The term relative refers to a person with whom the patient considers themself to have a close relationship (The National Board of Health and Welfare, 2021). Therefore, the patients suggested to who they had a close relationship and thus be eligible for an interview and forwarded contact information to the research team. The relatives were contacted by phone and given information about the study. Those who were willing to participate were to decide the time and place for the interview in order to create a calm and safe environment with as little risk of being interrupted as possible (Patton, 2015; Polit & Beck, 2021). Written consent was obtained from both the patients and their relatives.

### 3.3 Data collection

The interviews were conducted in a secluded room at the psychiatric clinic (2), in the relatives homes (3) or by phone (7) during November 2021 to January 2022. Before the interview, there was time to establish contact and take part in informal conversations and to give the informant the opportunity to raise questions. All relatives signed a written informed consent. The data were collected through individual semistructured interviews and conducted by HR and LW. The interview guide was designed in collaboration with the research team and consisted of open‐ended questions, as they tend to provide more detailed and spontaneous answers, which was considered appropriate for the purpose of the study (Patton, 2015). The questions asked were “How do you experience Brief admission as intervention for your relative?” They were also, for example, asked: “Can you tell if there is anything that you think can be developed with BA?” The question guide was supplemented with asking the relative to develop their answers, or with additional questions such as “Can you tell me more?”—so‐called probing questions (Patton, 2015). The interviews were recorded as digital audio files and then transcribed verbatim using a transcription guide (McLellan et al., 2003), to facilitate subsequent analysis. The interview had a median length of 40 min and during the last interviews no new data appeared and can thus be seen as a saturation of data. After the interviews, there was time for reflection, if required, by the informants. Two test interviews were conducted, and as no changes needed to be made, these interviews were included in the analysis. Participants were given information about setting aside approximately 60 min for the interview (Polit & Beck, 2021).

### 3.4 Data analysis

The results were analysed based on a conventional content analysis (Hsieh & Shannon, 2005). The interviews were read several times with the aim of creating an overall picture of the collected data. To find and highlight exact words in the text that capture key thoughts or concepts related to the aim, the transcripts were read word by word by all authors. Then, codes were marked and labelled as close to the original transcripts as possible. Codes with similar meaning were sorted into subcategories. The content in each subcategory was sorted into a smaller number of categories. Finally, definitions for each category and subcategory were developed. All authors agreed on how to categorize subcategories and categories. The name of the categories was derived from data, not from pre‐conceived categories, and thus an inductive approach was applied (Patton, 2015). The generated subcategories and categories are presented in a table (Table 1) (Hsieh & Shannon, 2005). Quotations for each category were identified from the transcripts to report the findings and strengthen the trustworthiness of the results (Polit & Beck, 2021). As a final step of analysis, one of the co‐authors read excerpts of the raw data to validate the content of the categories (Patton, 2015).

**TABLE 1:** An overview of the categories and subcategories produced using Hsieh and Shannon (2005) conventional content analysis

Category	Subcategory
Breathing space	Reduced anxiety Time for something else
Personal responsibility	Maintain skills Promotes responsibility
Structure	A predictable form of care A developable form of care

### 3.5 Ethics

The study has been approved by the Regional Ethical Review Board in Linköping, Sweden (Dnr: 2021‐04567) and was performed in accordance with the Declaration of Helsinki (World Medical Association, 2013). By obtaining informed consent from both the patient and their relatives, the idea was to avoid the patient feeling “that someone is talking over their head”. There is always a risk of provoking reactions in an interview situation. At the same time, in this case, a “silent group” is given the opportunity to express their thoughts and experiences. There was a readiness to handle emotional expressions, as the interviewers were nurses with many years of experience in psychiatric work. Both patients and relatives were informed that regardless of participation and experiences of BA, their future care would not be affected. Data are presented at a group level, which means that an individual informant should not be able to be recognized based on the descriptions.

## 4 RESULTS

The sample consisted of 12 relatives, of whom eight were women. They were aged between 22–75 years (mean age of 46). Their relationship to the patient varied and consisted of five mothers (one engaged), one grandmother, three spouses, one friend and one sibling.

The result consists of an overaching category: “Hope for the future” followed by three categories “Breathing space”, “Personal responsibility” and “structure” which are distinct from each other (Table 1). In each category, the varying experiences are highlighted in subcategories. In the results, the descriptions of the subcategories are illustrated by quotes from the participants, with the interview number of participants in parentheses.

### 4.1 Hope for the future

Brief admission creates opportunities for relatives to have time and space to take care of themselves, while at the same time BA creates conditions for the patient to live as normal a life as possible. BA shifts focus from relatives taking responsibility and constantly watching over the patient, to the patient him/herself taking responsibility for their own well‐being and care seeking. The clear structure of BA also contributes to a more easily planned and predictable everyday life with hope for an improved future for the whole family. However, the hope is conditional on BA being available when needed.

### 4.2 Breathing space

The knowledge that the patient has access to BA was experienced by relatives to provide respite and reduced anxiety. BA contributed to the patient being able to get help in a simpler way and at an early stage, which was described as giving time for other things.

#### 4.2.1 Reduced anxiety

Relatives describe a general feeling that had followed them for a long time, an anxiety that the patient will be mentally impaired, as their condition had previously led to self‐destructive or suicidal actions. After the patient has gained access to BA, relatives express reduced anxiety, as BA is easily accessible in comparison with usual psychiatric inpatient care. BA requires that the patient themselfs calls the department that handles BA and expresses the desire for care. The experience of relatives is that at the times the patient has received help at an early stage of the deterioration, the downward spiral of deterioration has been able to be stopped. The reduced anxiety of relatives is also based on the knowledge that the patient is in a safe place with access to care. It appears from the statements that only the knowledge that the patient has access to BA has reduced the relatives' concerns. The abovementioned factors contribute to giving relatives the opportunity to relax in a situation that would otherwise have been stressful for both the patient and relatives:

The security above all, I think, that she is under surveillance … she is in a place where she can still go out … she is not locked up. I do not think it will be as much anxiety for me … It will be a security for me that allows me to relax more. (P5)

#### 4.2.2 Time for something else

When the patient is not feeling well, relatives describe that this occupies their entire everyday life. They describe that they cannot think of anything other than the risk of the patient injuring themselfs. It also emerged that this led to their own health being left behind. With BA, relatives describe that they have been able to take care of themselves and let go of responsibility for a while:

Yes, then I feel better… then he can call and say ‘yes mom … now I do not feel well … I will go to bed a couple of days.’ Then I know that I can kind of let go and concentrate on myself a little. (P3)

There are relatives who experience difficulties in giving the remaining family members the attention they need. They describe situations where several family members do not feel well and where they previously had to prioritize the patient to avoid a deterioration, which meant that other things were left behind. With BA, relatives describe that they have been given time for the remaining family members at the same time as the patient has received care in the ward:

I have two more boys and one is only 14 years old and he tries to make himself invisible, especially in hard times … so that we do not have to care about him as well; and it is not healthy for him to make himself invisible just so that we can cope. With BA I have been able to dedicate that time to him instead. (P9)

### 4.3 Personal responsibility

The main finding of this category is that this form of care makes it possible to maintain skills, even during inpatient care. Relatives said that the patient had begun to take personal responsibility and seek help in time, compared with before access to BA.

#### 4.3.1 Maintain skills

Relatives describe that BA enables patients to maintain skills in everyday life, such as going to work, continuing to see their psychologist, meeting friends and spending time with family. Previous experiences with long care periods in conventional psychiatric inpatient care are described as contributing to the loss of skills that have taken a long time to rebuild:

A long time of hospitalization means that so much of her skills are lost and she is forced to start all over again from the beginning … and it takes so much effort to rebuild. (P9)

It takes a lot of energy and time for both the relative and the patient to regain these skills after long care periods. The patient has many times worked on the abovementioned skills through many years of therapeutic care that have been tough for both the patient and relatives. BA is thus described as a form of care that helps the patient to maintain the skills that have been developed:

It is very good that she can work two hours a day, even when she is hospitalized. She has a job she enjoys very much too, and she is educated and such. So it's good for her to come to work. (P11)

#### 4.3.2 Promotes responsibility

Relatives experience that BA is a form of care that allows the patient to become hospitalized, even where there is not an acute danger to the patient's life. It is experienced positively, as the patient does not have to exaggerate a deteriorating mood in the psychiatric emergency room to be admitted:

Now that she goes in … I feel the feeling of security that she actually comes in before she gets so really bad … I calm down. (P5)

Being cared for according to BA means that the patient is cared for in a freer way in comparison with the usual psychiatric inpatient care. The freer form of care described by relatives has contributed to the patient themselfs being able to make decisions about what concerns their own health. Relatives feel that the BA promotes the patient's ability to take responsibility for their health and helps them get to know their early signs of deterioration and to take responsibility for taking help in time:

The concept of going in yourself and making decisions when it's tough is good; I think you learn a lot from it. That you must take responsibility for your own health … That you can go to a place where you get help. (P5)

Brief admission makes it possible to receive care at an early stage, which contributes to a cancellation of the deterioration. Relatives experience that care at an early stage contributes to the patient taking responsibility for getting help before the health has become so impaired that they no longer have the ability or willingness to ask for help. Deciding when care is needed and deciding when inpatient care is no longer needed was felt to strengthen the ability to take responsibility. Most of the relatives said that the patient could remain in the ward during the usual psychiatric inpatient care when they themselves wanted to go home. It appears that in some cases it has contributed to a fear in the patient and relatives to seek care in conventional psychiatry. BA is therefore considered to be simpler in that aspect:

The thing about the hospital horror came … that she was forced to stay. (P4)

It also emerged from relatives that since the patients had gained access to BA, self‐harming behaviour and suicidal actions may have been avoided, as the patient received care at an early stage and had to take responsibility for when they needed care. Relatives state that previous deteriorations in the patient could have escalated, which meant that the patient had acted self‐destructively and needed police or ambulance transport to the psychiatric emergency department:

Before, there have been many suicide attempts, pickups by ambulance and police ambulance when it has been at its worst. … And now that he can choose this for himself when he feels worse, it is very safe that it exists. (P3)

### 4.4 Structure

This category illustrates that relatives experience that BA is a predictable and freer form of care, and therefore does not affect relatives in the same way as conventional psychiatric care. At the same time, it is a developable form of care where the lack of care places is perceived as a problem that makes it difficult to seek help at an early stage.

#### 4.4.1 A predictable form of care

The care time during BA is limited to one to 3 days. It is experienced positively that the time is pre‐determined, as relatives know when the patient comes home again. Among children BA was experienced as beneficial as they knew when the parent should come back home again. Traditional care can otherwise mean that the patient is away from home for long periods. BA is a freer form of care and which is described as something that facilitates everyday life, as the patient can move freely between home and the ward. This contributes, for example, to the patient being able to stay at home until the children go to bed and then go to the ward to be there during the night, when their mood may be perceived as worst for the patient. During BA, any medication adjustments were handled by the patient's outpatient doctor. This was experienced positively because previous experiences have been that outpatient care planning is not followed if the patient is admitted to a ward, and where the patient then tends to receive more and more medication:

I also think … that it will be easier for the children if they notice that mom is only in for two or three days … instead of not knowing … will she come back tomorrow or will she come back in August … October, November? (P1)

Relatives describe negative experiences when seeking care at the psychiatric emergency department. They describe uncertainty in whether the patient will be admitted or not, at times relatives believe that the patient needs help. The patient may need to exaggerate a deteriorating mood, by expressing suicidal thoughts and plans, to be admitted. It also emerged that relatives describe that the patient experienced a feeling of being ignored in their bad mood at the psychiatric emergency department, which made it difficult for relatives to motivate the patient to seek the usual psychiatric inpatient care. After the patient has had access to BA, it is described that it has been easier to motivate admission, because the structure with BA does not entail an uncertainty or risk of nonchalance that may otherwise be difficult to handle:

I think it's a great form of hospitalization because it's not like climbing a mountain. You do not have to go through the psychiatric emergency room and that uncertainty … will I get help or not? (P9)

Relatives appreciated the ward where BA is offered and described a calm over knowing which ward the patient will be cared for and who works there. Together with the patient, they had positive experiences of the ward, which contributed to a calm. In previous admissions via the psychiatric emergency department, the uncertainty about where and if the patient will be admitted has been a cloud of concern, as relatives know that the patient is not feeling well in certain psychiatric wards:

The care is very different, depending on which ward has a place, at that very moment, so that instead you can know, that if you apply for this, well then, it is this ward and then you know a little what kind of staff it has … to know that feels good I think. (P6)

If it turns out during BA that the patient is in need of extended care, the patient will be helped to seek care at the psychiatric emergency department together with staff from the ward. For example, it may be relevant if the patient self‐harms during the care period, expresses imminent suicide plans or is deemed to need further care after 3 days. This part of the structure with BA is perceived as security for the relatives:

It's better with BA, then, if you feel really bad you can transform it … Think about whether this is enough. Then she is already in place with professional people. (P9)

The limited care time was described as contributing to the patient not being as inclined to engage in the condition of other inpatients. Previous experience from the relatives has shown that long care periods have contributed to the patient being largely involved in other patients' well‐being. The commitment to others has been experienced by relatives as affecting the patient in a negative way:

An unhealthy grouping among patients. Often when you are hospitalized longer … you affect each other negatively. (P1)

#### 4.4.2 A developable form of care

Brief admission is generally appreciated, but aspects emerged that can be improved based on information for relatives, self‐responsibility and shortage of care places. The knowledge about BA varied, requests for information for relatives emerged, and the desire to visit the ward, see the room intended for BA, and meet the staff, was expressed. This was in order for relatives to gain increased knowledge about BA and be able to form their own opinion about the form of care:

It's if you can follow him when he is admitted so that you can see how it is and how it works, so you can understand, because now I do not know how it is really. (P2)

Someone experienced BA as meaningless, and that the patient did not receive the support she needed, or was unable to take on the responsibility that the form of care pre‐supposes. There was a disappointment that the staff did not act when the patient needed increased support and supervision. In this context, routines for daily conversations during BA were requested:

The experience I have, it's just negative. I probably think that it could have been positive … but I'm not sure it's right for her either. But I also think, if you are enrolled … in a team … then they, around her, should be able to say that, ‘No, this is not for you’. (P7)

The lack of care places was perceived as a limitation with BA, and being denied a place made it difficult to seek help in time. If patients had been denied several times, the joy of being offered this form of care disappeared and made it more difficult to apply. That BA is not available around the clock was experienced as a limitation. Furthermore, doubts were described as to whether the care environment in the ward could be a limitation with BA, as the patient is cared for in an environment he or she previously felt bad about:

It is difficult to get in on time if there are no places. And I know the last few times … she has called many times before she got a place and then she had already gotten worse. (P12)

## 5 DISCUSSION

To our knowledge, there are no previous studies that shed light on relatives of people with BPD and self‐harming behaviours in relation to their experiences of BA. Thus, the results of this study contribute important and new knowledge about BA viewed from a broader perspective. The main result of this study illustrates that access to BA inspired hope for the future and allowed relatives to let go of their worries about the patient and instead devote their time to the rest of their family and themselves. The fact that BA is limited to 3 days provides a structure, and further knowledge about when the patient would come home made it possible to plan everyday life based on that. Relatives who were not familiar with the purpose of BA and the patient's individually written contract, perceived the form of care as meaningless. Refusal of BA due to lack of space constituted an obstacle to a well functioning form of care. The results showed the relatives' experiences of BA, but also their reflections on and mirroring of the conventional psychiatric inpatient care, which they are very familiar with. A very problematic picture emerges in relation to conventional care. Patients have had to exaggerate their symptoms to be hospitalized, and then deteriorate during the often long period of care. In light of this, BA appears to be a credible alternative, although it can be developed and improved.

Relatives in this study describe that, before the patient had access to BA, they have felt a significant concern when the patient's mood deteriorated. They describe it as all their time and energy was spent worrying and helping the patient who was not feeling well. Relatives of people with mental illness tend to neglect their own interests and health as they feel anxious and a need to be there to help (Weimand et al., 2013). Ekdahl et al. (2011) describe that relatives of patients with BPD often have an energy‐intensive readiness and a fear that their actions will provoke the patient to act self‐destructively. Remaining family members can then end up in the shadows, which creates feelings of guilt and a feeling of not having enough (Radfar et al., 2014). The results of this study show that BA gave relatives a breathing space and time to prioritize their own interests or help others in the family not feeling well. It also emerged that relatives perceived BA as a positive form of care that does not affect the children to the same extent. When the children knew when the parent would return home, their uncertainty diminished. Children who live with a parent who suffers from mental illness may experience anxiety about the unpredictable and anxious situation they live in. The child can then try to compensate for this by taking on a caring role and taking over responsibility for household chores, which can affect the child negatively (Hedman Ahlström et al., 2009). By using BA, the child is less affected, as the parent can still be present, even during inpatient care. The fact that the children are not affected to the same extent is considered an important factor to point out from the relatives' perspective about the BA form of care. In this study, it emerged that relatives take great responsibility for the patient and have difficulty focusing on anything other than their mood. Many relatives of patients with BPD describe the fear that something terrible will happen that may result in the patient's suicide (Ekdahl et al., 2011). In addition, it is considered of great importance to avoid a deteriorating mood that can lead to the patient being exposed to risks. BA is also referred to as a tool for preventing suicide attempts (Arnold et al., 2021). The fear that the patient will hurt themselves or take their own life during a deterioration is something that recurs among relatives in this study, and access to BA therefore contributed to relief. Living with someone with mental illness is described by close relatives as a great responsibility (Radfar et al., 2014). Relatives estimated that BA gave the patient the opportunity to apply at an early stage, and that further deterioration could be avoided. Similar results have been described by nurses and patients (Eckerström et al., 2019; Enoksson et al., 2021).

Increased patient responsibility and care at an early stage can thus be perceived as significant and helpful factors from the perspective of both the patient (Eckerström et al., 2020; Enoksson et al., 2021), healthcare staff (Arnold et al., 2021; Eckerström et al., 2019) and, as found in this study, relatives. Relatives in this study said that they promote the patient's ability to take responsibility for, and be observant of, early signs of deterioration, in that it is the patient who has control over enrollment and discharge, which has also emerged in other studies (Ellegaard et al., 2017; Enoksson et al., 2021; Helleman et al., 2014a, 2014b). This is something that could be linked to the fact that BA is a nursing intervention that strengthens the patient's autonomy, which is an important part of person‐centred care (Ekman et al., 2011; Gabrielsson et al., 2015). However, based on our result, family‐centred care could be given more attention in BA. The results of this study indicate that relatives experience an improved life situation by giving time to their own interests and that the structure of the form of care provides an opportunity to plan everyday life. Eckerström et al. (2022) report in their study that the majority of patients who use BA estimate a higher quality of life in comparison with before BA (Eckerström et al., 2022). No concrete measurements about the life situation of relatives have been carried out in this study and is why it would be valuable to further investigate this in future studies. The well‐being of relatives is important, as they have a supportive role for the patient (Sharma et al., 2016; Ten Have et al., 2016).

The majority of relatives appreciated the freer form of care, with a limited number of care days, because the patient's ability to, for example, take care of the children and go to work is maintained. From the patient's perspective, some experience concerns about the limited number of care days (Ekman et al., 2011). These varying perceptions can be understood from the fact that patients use BA to get a break from everyday life, especially during stress (Enoksson et al., 2021), while relatives, as mentioned above, find it positive that the patient returns home quickly to help out with daily chores. Relatives of people with BPD experience more suffering than relatives of people from other diagnostic groups (Bailey & Grenyer, 2014), and many suffer from anxiety and depression (Bailey & Grenyer, 2014; Ekdahl et al., 2011), which makes them even more exposed to mental diseases. BA may, although it is structured and restricted to a limited number of care days, seem to create space for relatives to recover. Although some patients experience a limitation in the number of care days during BA, it is perceived as helpful in reducing stress (Enoksson et al., 2021). This emerged from relatives in this study, which means that BA should not only be considered helpful from a patient perspective but also from the relatives' perspective.

In this study, relatives highlight that refusal of BA due to lack of space made some patients not so willing to use it again. This contributed to an increased concern among relatives that the patient would self‐harm and led to frustration over lack of BA places. When based on person‐centred care, care should be designed based on the patient's individual needs (Gabrielsson et al., 2015). From the patients' perspective, a disappointment arose if the room was occupied, making it difficult for them to call the next time (Helleman, Goossens, et al., 2018; Helleman, Lundh, et al., 2018). The knowledge of quick help is fundamental and constitutes a security in BA (Eckerström et al., 2020; Strand & von Hausswolff‐Juhlin, 2015). Relatives in this study also mention the lack of space as an obstacle to achieving this security, as a refusal can lead to the patient acting self‐destructively (Helleman, Goossens, et al., 2018; Helleman, Lundh, et al., 2018). The lack of space then becomes an obstacle to working in a person‐ and a family‐centred way, because then both the patient's and relatives' autonomy and participation are hindered by organizational factors (Ekman et al., 2011; Feinberg, 2014; Millenson et al., 2016).

The results showed that relatives can perceive BA as meaningless, and a structure with daily conversations was requested. How the agreement is designed, and how involved the patient and relatives are, can play a role in the experience of the form of care, as the patient themself is the one who communicated wishes about what should happen during the care period (Eckerström et al., 2019; Helleman et al., 2014b). The results showed that some relatives did not know that there was an individual agreement, which could explain why the form of care is perceived as meaningless. Some relatives wanted more information and wanted to participate in BA, while others wanted to be out of care. In psychiatric care, it appears that relatives are not involved or did not receive information to the extent they desired (Cree et al., 2015). Involving relatives can be a challenge for care because some patients do not want to have them involved for various reasons. However, it emerged in the results that all relatives do not want to be involved as they had, before BA, put in a lot of effort and pushed hard in order for the patient to receive care. According to the Patient Act SFS, 2014:821 (Ministry of Health and Social Affairs, 2022), close relatives must be involved as far as possible and given the opportunity to communicate their opinion and have the opportunity to influence the implementation of care. The Patient Act requires that health care acts in a person‐centred way and involves close relatives. The relatives play an important role in the patient's life and the patient's opportunity for recovery, as they possess knowledge about the patient's need of care (Juntunen et al., 2018), and involving relatives in care is also one of the basic principles in person‐centred and family‐centred care (Feinberg, 2014; Gabrielsson et al., 2015; Millenson et al., 2016). Studies show that patients with BPD, without relatives present, are at risk of developing more severe symptoms related to their diagnosis (Ten Have et al., 2016). Many relatives feel excluded from care (Ekdahl et al., 2011). Excluding relatives is something that must be passed on to history. Relatives should be recognized as a partner, as it is likely to be gainful for all parties (Juntunen et al., 2018). Today, BA does not involve relatives; by asking the patient if relatives may be present when an agreement is written can be one way to include relatives.

### 5.1 Limitations

When using the conventional content analysis, there is a risk that the authors fail to identify important parts of the respondents' statements (Hsieh & Shannon, 2005). The reliability of qualitative descriptive research is based on how well the authors managed to exclude irrelevant data and include and categorize relevant data according to what the respondents shared in the interviews (Polit & Beck, 2021). To increase reliability, the results have been strengthened with quotes from the transcripts, and throughout the analysis process all authors collaborated with each other so to ensure no relevant data was missing. First and last authors have solid experience of analysing qualitative data, which can also increase credibility (Patton, 2015).

Although BA is currently offered in several different countries with the common denominator “self‐referral admissions to psychiatric inpatient care”, the execution differs depending on how the psychiatric care is structured locally (Eckerström et al., 2022; Enoksson et al., 2021; Helleman et al., 2014a). Therefore, the transferability of the results may be limited. A clear description of the relatives, the context in which the study took place and of the research process, are given in order to make it possible for the reader to decide whether the results are transferable to another context (Patton, 2015). Most of the interviews were conducted digitally. Face‐to‐face interviews are considered to be the most optimal in qualitative research, as data can thereby be enriched with body language and emotional reactions among participants, which cannot be obtained through telephone interviews (Polit & Beck, 2021). In qualitative research, however, telephone interviews are described as a good alternative as they are less time‐consuming and provide more flexibility at, for example, large geographical distances (Opdenakker, 2006). The possibility of the flexibility of being able to interview despite geographical distances were considered as important factors in obtaining relevant data in this study. Of the relatives, only four were men. In the selection process, the gender perspective was not taken into account as this was not considered to be relevant for the purpose of the study (Curtis et al., 2000). However, in future research, it would be interesting to shed light on whether there are differences in perceptions of BA seen from a gender perspective. It was surprisingly easy to recruit relatives to the study and they all expressed gratitude for participating. Previous studies illustrate that social stigma is the most common and challenging burden among relatives of people with BPD (Meshkinyazd et al., 2021), and many relatives feel excluded from health care (Ekdahl et al., 2011). As mentioned earlier, the relatives who participated in this study had great amounts of knowledge of their close ones' needs and were familiar with how existing health care works for them (Juntunen et al., 2018). To ensure validity, a semistructured interview guide with open‐ended questions was used. This is a pre‐requisite to catch the informants' thoughts, experiences and perceptions about parts of the world about which they are talking, in this case BA (Patton, 2015).

## 6 CONCLUSION

When BA functions and is available in an early stage to prevent self‐harm, relatives said that they had a breathing space and time for recovery. Furthermore, BA contributes to relatives daring to let go of the responsibility they previously took over the patient, which promotes the patient's autonomy and relieves the burden of the relatives. Not all relatives had the information they wanted about BA. By asking the patient if relatives can be involved when an agreement is written, relatives would be given better conditions to receive information and be involved, which could also promote a person and family‐centred approach in psychiatric inpatient care. Furthermore, it emerged that the children are less affected during BA than usual care, as it is clearly time‐limited in its approach. This led us to the conclusion that BA is a form of care that should not only be considered helpful from a patient's perspective but also from a relative's perspective.

## 7 RELEVANCE TO CLINICAL PRACTICE

For relatives to trust and feel secure with BA, care beds must be available when needed. As relatives have an important role in the patient's life and want to be supportive, they need to be given information and the opportunity to be included in the BA concept. Relatives described an improved life situation for themselves and for the children, but larger quantitative studies are needed to explore this further.

## FUNDING INFORMATION

This work was supported by the Department of Psychiatry, Ryhov County Hospital, Jönköping.

## CONFLICT OF INTEREST

The authors report no conflicts of interest.

## ETHICAL APPROVAL

Relatives of patients who have access to BA shared voluntarily their experiences during an interview. The authors have confirmed that all authors meet the ICMJE criteria for authorship credit (www.icmje.org) as follows: (1) substantial contributions to the conception and design of or acquisition of data or analysis and interpretation of data, (2) drafting the article or revising it critically for important intellectual content, and (3) final approval of the version to be published.

## Data Availability

Data available on request due to privacy/ethical restrictions.
